# Petri net modeling and simulation of post-transcriptional regulatory networks of human embryonic stem cell (hESC) differentiation to cardiomyocytes

**DOI:** 10.1515/jib-2024-0037

**Published:** 2025-06-23

**Authors:** Aruana F. F. Hansel-Fröse, Christoph Brinkrolf, Marcel Friedrichs, Bruno Dallagiovanna, Lucia Spangenberg

**Affiliations:** Laboratory of Basic Stem Cell Biology, Carlos Chagas Institute, Oswaldo Cruz Foundation (FIOCRUZ/PR), Curitiba, Brazil; Division of Immunobiology, Institute of Immunology, Center for Pathophysiology, Infectiology and Immunology, Medical University of Vienna, Vienna, Austria; Faculty of Technology, Bioinformatics/Medical Informatics Department, Bielefeld University, Bielefeld, Germany; Bioinformatics Unit, Pasteur Institute of Montevideo, Montevideo, Uruguay; Departamento Basico de Medicina, Hospital de Clinicas, Universidad de la República (Udelar), Montevideo, Uruguay

**Keywords:** cardiomyogenesis, gene regulatory networks, microRNAs, untranslated regions, computer simulation

## Abstract

Stem cells are capable of self-renewal and differentiation into various cell types, showing significant potential for cellular therapies and regenerative medicine, particularly in cardiovascular diseases. The differentiation to cardiomyocytes replicates the embryonic heart development, potentially supporting cardiac regeneration. Cardiomyogenesis is controlled by complex post-transcriptional regulation that affects the construction of gene regulatory networks (GRNs), such as: alternative polyadenylation (APA), length changes in untranslated regulatory regions (3′UTRs), and microRNA (miRNA) regulation. To deepen our understanding of the cardiomyogenesis process, we have modeled a GRN for each day of cardiomyocyte differentiation. Then, each GRN was automatically transformed by four transformation rules to a Petri net and simulated using the software VANESA. The Petri nets highlighted the relationship between genes and alternative isoforms, emphasizing the inhibition of miRNA on APA isoforms with varying 3′UTR lengths. Moreover, *in silico* simulation of miRNA knockout enabled the visualization of the consequential effects on isoform expression. Our Petri net models provide a resourceful tool and holistic perspective to investigate the functional orchestra of transcript regulation that differentiate hESCs to cardiomyocytes. Additionally, the models can be adapted to investigate post-transcriptional GRN in other biological contexts.

## 1 Introduction

Cardiovascular diseases are the world’s most common cause of mortality, summing about 17.9 million deaths each year [1]. It is well known that human adult cardiac tissue expresses a low regeneration rate due to low proliferation from cardiomyocytes [2]. Thus, an alternative to heart transplant in addressing cardiac failure is the regeneration from parts of the malfunctioning tissue with new healthy cardiomyocytes. In this context, stem cells have potential of being used in the field of regenerative medicine [3]. Because of the cells’ ability to self-renew and differentiate, there is particular interest in the treatment of cardiovascular disease [4], [5], [6]. Pluripotent stem cells are good study models for cardiac regeneration [7], [8], [9]. In addition to having therapeutic prospects [3], 5], they can differentiate to cardiomyocytes similarly to the embryonic heart development [10], [11], [12]. During cardiomyogenesis, there is complex transcriptional and post-transcriptional regulation, in which many key players are involved, including coding (messenger RNAs, mRNA) and non-coding RNAs (ncRNAs), such as long non-coding RNAs (lncRNAs) and microRNAs (miRNAs) [13], [14], [15].

Our group has previously shown that differentiation of human embryonic stem cells (hESCs) to cardiomyocytes involves post-transcriptional regulation and translational control. The sole recruitment of transcripts, coding and non-coding, to the ribosomal complex contributes to the cardiomyogenesis process [13], 14]. Additionally, we have also observed that alternative polyadenylation (APA) can have an impact in the differentiation of cardiomyocytes [16]. This mechanism can generate many isoforms of the same gene, differing only in the length of the 3′ untranslated region (3′UTR), without changes in the coding sequence [17]. Consequently, changes of 3′UTR length can impact binding of regulatory elements, such as microRNAs. These small ncRNA of about 18–22 nucleotides can inhibit gene targets through target sites usually located on the 3′UTR of the mRNA [18]. To unravel the implications of this complex post-transcriptional regulation, we have previously constructed Gene Regulatory Networks (GRN) to elucidate how APA isoforms, their changing 3′UTR lengths and miRNA targets affect the dynamic gene expression landscape during cardiomyogenesis differentiation of hESCs [16].

The construction of GRNs provides a comprehensive understanding of the complex interplay between various relevant transcripts during cardiomyogenesis that is not easily feasible *in vitro*. Computational modeling of GRN can deepen our understanding of biological systems by dealing with a large amount of data and the calculations to find the most relevant connections between biological elements [19], 20]. Moreover, modeling of GRN enables the investigation of gene interactions and the manipulation of gene expression *in silico*. This strategy can direct *in vitro* validations on predicted pathways and relevant signaling found in the model [21], 22].

A gene regulatory system can be represented as a GRN, which can be either an undirected or directed graph. In the former, connections between nodes have no particular direction. On the other hand, in the latter, the connections indicate a specific direction of interaction between the nodes [19]. Examples of applied undirected graphs are protein-protein-interaction networks, while biochemical pathways and GRNs are better modeled by directed graphs due to the nature of the reactions.

Some modeling approaches for directed graphs are Bayesian networks, Boolean networks, and Petri nets [20], 23]. Biological networks have been successfully computationally modeled by these approaches in the past, with Petri net methods standing out in this field [20], 22], [24], [25], [26], [27], [28].

Bayesian networks deal with conditional probability to build the connectivity between nodes. Since multiple tests are needed until the best model is found, this method requires a high computational processing capacity [23]. As for Boolean networks, its interactions are represented by Boolean functions that calculate the state of a gene by activation of other genes [23]. Since genes can show two states, activated or inactivated, the Boolean method does not account for intermediate expression thresholds [23].

As for Petri nets, invented by and named after Carl Adam Petri [29], they are a directed bipartite graph consisting of two types of nodes: places (drawn as circles) and transitions (drawn as rectangles). In the system, places represent the conditions whereas transitions represent the actions. Tokens are the system units, which can pass through the system depending on the pre-defined transitions, if the necessary criteria are met for the execution of that action [30]. Adaptable, Petri nets allow the representation of large and dynamic systems in a simple and compartmentalized way, through the modeling of small subprocesses [24]. The network allows the simulation of gain or loss of function, an interesting characteristic in the context of GRN [22], 26]. Additionally, it enables simulations of large networks, an advantage over Bayesian and Boolean networks [20], 24].

There are several extensions of the basic Petri net formalism that enhance its modeling capabilities. For example, incorporation of stochastic transitions to represent probabilistic behavior. Functions can be used as arc weights and transition properties, allowing the modeling of complex dynamic networks [31], [32], [33].

In this context, VANESA [34] is an open-source hybrid modeling, transformation, and simulation environment for biological networks and Petri nets. It enables the reconstruction of a variety of biological networks. Supported biological node entities are enzyme, DNA, mRNA, and miRNA, among others. Biological networks can be automatically transformed into Petri nets based on a set of customizable user-defined rules with predefined parameters. This allows the representation of various types of biological systems and their transformation to meaningful Petri nets. Complex networks can easily be created, manipulated, and visualized within the same software. Also, simulations can be run, and gain or loss of function can be assessed. Finally, a connected data warehouse that includes repositories, such as KEGG pathways [35], facilitates further data integration.

Petri nets have been used to represent a variety of biological networks and contexts, both in health and disease [22], 25], [36], [37], [38], [39], [40]. In terms of cardiomyocytes, they have been applied to model cardiomyocytes in pro-apoptotic signaling pathways [41] and the *Wnt/* *β* *-catenin* signaling pathway [42]. As for stem cell’s differentiation, the haematopoietic GRN was previously modeled [43] to evaluate their self-renewal capacity [44]. In other biological scenarios, miRNA inhibition have been modeled in the epidermal growth factor receptor (EGFR) signaling pathways [45] and in the context of disease state and treatment [25], 46].

However, to our knowledge, Petri nets were still not applied in the context of cardiomyocyte differentiation, especially in a GRN that addresses post-transcriptional regulation of alternative polyadenilation, polysomal recruitment, and miRNA targeting.

Here, we have deepened our understanding of the previously constructed post-transcriptional GRNs of cardiomyogenic differentiation of hESCs [16] by modeling them as biological networks and automatically transformed them to Petri nets using VANESA. All the three stages of cardiomyogenesis were modeled. Starting from the mesodermal stage (day four – D4P), to progenitor (day nine – D9P), and final day of beating cardiomyocytes (day fifteen – D15P). The networks were modeled taking into account the expression of alternative polyadenilated transcripts and the specific expressed miRNAs that target them. Inhibition of miRNAs was included, which regulated specific transcript production. Beyond that, we have simulated miRNA knockout and its consequences in the network. Together, the resulted Petri net models of the cardiomyogenesis post-transcriptional GRN provides a valuable insight into understanding how cardiomyogenesis is finely tuned beyond transcription. We show that expressed miRNA can influence not only gene targets, but specific alternative isoforms, and this regulation can be manipulated *in silico*.

Finally, these models are a versatile tool that can be adapted to other biological contexts. The Petri nets are particularly relevant in those cases where investigation of miRNA regulation on a transcriptomic level will benefit from *in silico* manipulations of miRNA expression.

## 2 Materials and methods

### 2.1 Gene regulatory networks

The post-transcriptional gene regulatory networks that are used for the modeling and simulation of the Petri nets were previously constructed by our group and are described in more thorough detail in [16].

#### 2.1.1 RNA sequencing analysis

In summary, the transcriptome data that is used to generate the GRN was obtained from the RNA sequencing followed by polysome profiling of the hESC line hES-NKX2-5eGFP/w differentiation to cardiomyocytes. This was previously carried out by our group and is fully described in [13].

In detail, hESCs were differentiated to cardiomyocytes. Polysome profiling was carried out at specific time points of hESCs differentiation: day zero (D0), day one (D1), day four (D4), day nine (D9), and day fifteen (D15). With this technique, RNA was separated into two fractions: transcripts that are bound to polysomes (P) and the transcripts that are free from polysomes (L). From the polysome-bound transcripts fraction (P) of all days of cardiomyogenesis, bulk RNA sequencing was performed, generating the samples D0P, D1P, D4P, D9P, and D15P. Reanalysis of the RNA-seq data started with quality control with FastQC [47] and trimming of reads with Trim Galore (v.0.4.0) [48]. Alignment of reads was done with HISAT2 (v.2.1.0) [49] with the human genome version GRCh38 and the reads were counted with HTSeq (v.0.11.1) [50]. The differential gene expression analysis was done with DESeq2 (v.1.24.0) [51], comparing each day of cardiomyogenic differentiation (D1P, D4P, D9P, and D15P) to the pluripotent stage (D0P). Differentially expressed genes were considered using an adjusted p-value cutoff of 0.05 and log2FoldChange (log2FC) cutoff of |2|. Normalization of reads to Counts Per Million (CPM) was carried out in R (v. 4.2.2.) [52].

#### 2.1.2 Alternative polyadenylation isoforms

The alternative polyadenilation (APA) isoforms and their respective 3′UTR start and end sites were identified using APAtrap [53] in each sample (D0P, D1P, D4P, D9P, D15P), as detailed in our previous work in [16]. The significant APA isoforms were considered by the adjusted p-value cutoff of less than 0.05 and percentage difference greater than 20 percent, which are parameters of APAtrap as provided in [53] (Supplementary Data). Only the APA isoforms derived from differentially expressed genes were kept.

#### 2.1.3 miRNA targets

The miRNA expression data was obtained from the miRNAome of cardiac differentiation of pluripotent stem cells with corresponding time points to the samples, obtained by Garate and collaborators [15]. Differentially expressed miRNAs were filtered considering the cutoff of log2FC of |2| and adjusted p-value of 0.05.

To predict the targets from miRNAs specifically on the 3′UTR sequence of the previously selected APA isoforms, first their 3′UTR sequences from the human genome version GRCh38 was retrieved. Then, the human miRNA seed sites from the repository of miRBase [54] were obtained. The prediction of the miRNA targeting specifically on the 3′UTR sequence from the APA isoforms was carried out with psRNATarget [55].

#### 2.1.4 Construction of the GRNs

For further investigation, one GRN was created for each day of cardiomyogenic differentiation (D1P, D4P, D9P, and D15P). However, in D1P, only two genes were differentially expressed and showed differentially expressed APA isoforms. Therefore, the D1P GRN was not further investigated.

For the other days of differentiation, the nodes of each GRN are the differentially expressed genes, the differentially expressed miRNA that targeted the 3′UTR regions, and the specific APA isoforms from the genes. The edges represent the logical connections between the nodes.

Each node has five attributes: its name, label, start concentration, log2FC, and color. The label defines the biological type (‘DNA’ for genes, ‘miRNA’ for miRNAs, and ‘mRNA’ for APA isoforms). The start concentration is the normalized read count as CPM. The log2FC values were included in the corresponding nodes of the graph object with the label ‘log2FC’. In the case that DNA had more than one mRNA isoform, the isoform that receives the concentration value is chosen randomly. The gene nodes received a blue color, whilst miRNA nodes received a purple color. The shade of the node color is set to a value reflecting its log2FC. If the log2FC value is higher than 2 (upregulated), it is set to a dark color code. If it is lower than −2 (downregulated), it is set to a lighter color code. All transcript isoforms received the default dark navy color.

Similar to the nodes, each edge has a label defining its biological type, which is set to ‘Physical Interaction’.

The data structure for a GRN is a graph object from the R package iGraph [56], which also provides the function “graph_from_data_frame”. In addition, this package was used to export each GRN as a GraphML file, which in turn can be imported by VANESA without loss of relevant information.

### 2.2 Generation of the Petri nets

Each constructed GRN was imported to VANESA as a biological network for further transformation, simulation, and analysis. The latest updates to VANESA, released as version v.0.5, were used.

For the investigation of the quantitative temporal behavior of each GRN, it needed to be transformed into a mathematical formalism that allows for such simulations. For this purpose, each imported biological network was automatically transformed to a Petri net using four designed transformation rules and the rule-based transformation provided by VANESA. Each Petri net is a functional Petri net (FPN) [57] with inhibitor arcs. FPNs with inhibitor arcs are a subset of hybrid functional Petri nets (HFPN) [58]. In general, VANESA supports extended hybrid Petri nets (xHPN) [59], a superset of HFPN, for Petri net modeling, transformation, and simulation.

#### 2.2.1 Design of the transformation rules

Four transformation rules were designed that allowed the transformation of each GRN to a meaningful FPN with inhibitor arcs. The default values of Petri net elements are: number of start tokens of a discrete place is 0, delay of a discrete transition is 1, and the regular arcs and inhibitor arcs weight is 1. In the following, the four transformation rules and their values differing from the default values are described.

**Figure 1:**
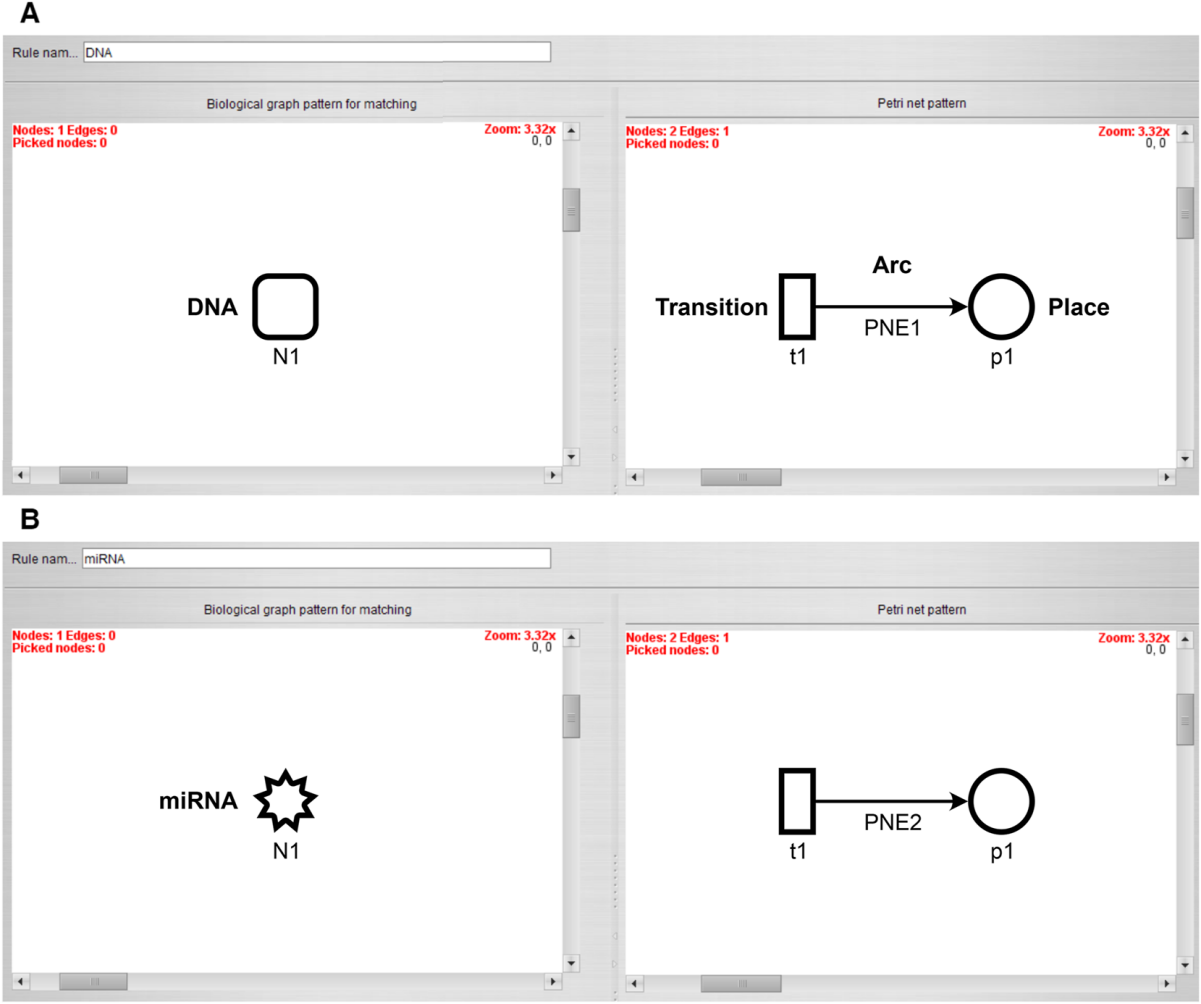
Transformation rules with the biological pattern on the left side and its Petri net representation on the right side. (A) Transformation of a DNA node and (B) transformation of a miRNA node. Both nodes are transformed to a place that is fed by a transition.

The first rule transforms a DNA node to a place which is fed by a transition, as shown in Figure 1A. The arc weight and the number of start tokens of the place are set to the start concentration of the DNA node.

Similarly, the second rule transforms the miRNA node to a place that is fed by a transition. The arc weight and the number of start tokens of the place are set to the start concentration of the miRNA node (Figure 1B).

**Figure 2:**
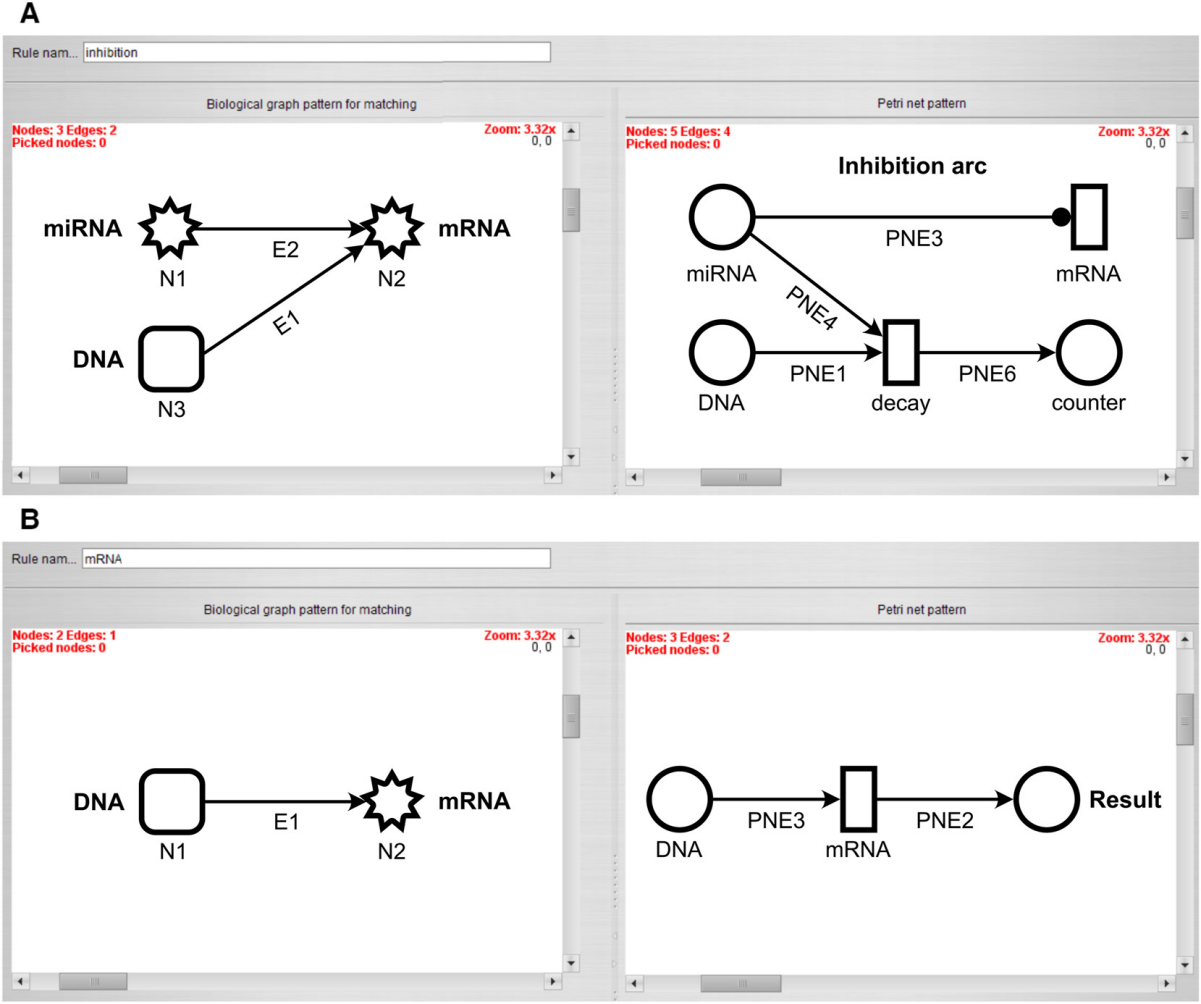
Transformation rules with the biological pattern on the left side and its Petri net representation on the right side. (A) Transformation of the inhibition and decay process by an inhibitor arc and a decay transition. (B) Transformation of the production of a mRNA by a DNA to a result place that is fed by the mRNA transition.

**Figure 3:**
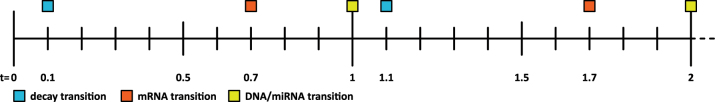
Section of the Petri net simulation timeline from time-point zero to two, representing the order of transition firings based on their defined delays.

The third rule represents the inhibition of miRNA over the mRNA isoform (Figure 2A). The mRNA node is transformed to a transition, and the miRNA node and DNA node are mapped to those discrete places created by the first and second rule. The miRNA place is connected to the mRNA transition by an inhibitor arc. Further, the miRNA place and DNA place are connected to a decay transition which is feeding a counter place. The arc weights of the arcs from and to the decay transition are set to a function that determines the minimum number of tokens of the miRNA place and DNA place. This function is evaluated during each step of the simulation. The delay of the mRNA transition is set to 0.7 and the delay of the decay transition is set to 0.1. This ensures that available miRNA first binds to DNA and only remaining miRNA might inhibit the mRNA transition. The timeline of transition firings based on these delays is visualized in Figure 3.

The fourth rule connects the DNA place created by the first rule with the mRNA transition created by the third rule with a regular arc, as shown in Figure 2B. Further, the transition is feeding a result place, named *GENE_TRANSCRIPT_FINAL* where GENE and TRANSCRIPT are the names of the parent gene (DNA) and transcript isoform (mRNA), respectively. The arc weights of both arcs are set to the number of tokens of the DNA place, and this number is evaluated during each step of the simulation.

It is ensured that all constants and functions evaluate to integers greater than zero by rounding up to the next integer. In addition, it is ensured that arc weights of arcs to a transition evaluate to a number greater than zero. This is archived by replacing each arc weight function *f* by max(*f*, 1) to avoid enabling of transitions with zero tokens in their pre-places.

Further, if a node of the biological network is represented by a Petri net node, its color code is reflected by the corresponding Petri net node.

For simplicity in the context of Petri nets, biological nodes that are mapped to Petri net nodes are referred to by their biological meaning. Hence, a place corresponding to a DNA node is referred to as a DNA place.

#### 2.2.2 Biological interpretation of the Petri nets

Each Petri net model that was automatically created by the transformation represents a GRN during the process of transcription. Transcripts of the involved DNA and miRNA are generated with a constant speed, given by their start concentrations, as outlined in the first and second rule. The start concentration of a biological node thus leads to a constant generation of tokens of its corresponding place in the Petri net. The third rule models the inhibition of mRNAs. Each mRNA is represented as a transition and thus treated as a process. Those processes are inhibited by available miRNAs connected to the particular mRNAs by inhibitor arcs. As long as there is miRNA present, the mRNA transition is not active.

During each step of simulation, first, the minimum of available miRNA and DNA are bound and removed by the decay transition. The counter keeps track of the number of bound DNA and miRNA for later evaluation. The functions assigned to the involved arcs as arc weights calculate those minimum values. Once a mRNA is not inhibited by any connected miRNA, it is active and processes all remaining DNA that is counted for later evaluation by the result places modeled by the fourth rule. This strict firing order is modeled by the delay of the involved transitions.

In general, the model assumes that during each step of the simulation: (1) transcripts are generated at a constant speed, (2) the presence of a single miRNA inhibits a mRNA process entirely, regardless of the number of connected miRNA and DNA nodes, (3) minimum number of available miRNA and DNA bind and decay first, and (4) all remaining DNA is then processed by the mRNA process.

### 2.3 Simulation of the Petri nets

VANESA uses an installation of OpenModelica [60] and the Petri net library PNlib [61] for the Petri net simulation. The Petri nets were simulated for 20 time units with the following settings: OpenModelica 1.24.4, PNlib 3.0.0, using a short model name, and 2,000 equations per file. The last two options were necessary to simulate the largest Petri net, D15P. The simulation of each Petri net for 20 time units was sufficient to observe its temporal behavior.

The interpretation of the simulation results was based on the transcript productions, namely the places called “GENE_TRANSCRIPT_FINAL” which contained the final number of tokens that were not sufficiently inhibited by the miRNAs and, therefore, would be expressed.

Adjustments of the model can be done by manipulating the biological network before its transformation to the Petri net is performed. A change of the start concentration of the miRNA or DNA node would for example reflect loss or gain of expression. Further, the generated Petri net can be adjusted by altering the transformation rules and by manipulating values of Petri net elements after its generation.

### 2.4 Visualization of the Petri nets and their simulation results

The generated Petri nets and their simulation results were visualized in VANESA. The generated Petri net automatically incorporated color coding to indicate transcript expression levels. This allowed quick interpretation of differential expression fold changes from the previous biological network analysis. The layout used for the visualization of Petri nets is an implementation of the GEM (short for *graph embedder*) algorithm [62].

To analyze the simulation of specific and relevant genes from the original biological networks, we focused on enhancing the visualization of the most relevant places and transitions. To achieve this, Petri net counter nodes that were not essential for interpretation, were excluded in visualization. The figures were exported by VANESA as PDF files. The biological networks and their corresponding Petri nets were saved as SBML files and the simulation results were exported as CSV files. All files are available as Supplementary Material.

## 3 Results

The results of the Petri net simulations are evaluated based on the accumulation of tokens in relevant places and compared against the differential expression analysis. First, this section describes the interpretation of the simulation results in general followed by a detailed analysis of selected network parts.

### 3.1 Interpretation of the simulation results

For the evaluation of the simulation results, pairs of genes of interest related to cardiomyocyte differentiation were pre-selected according to the previous research [16] and to common miRNA target regulation. Then, the gene pairs were evaluated regarding their differential expression fold change (log2FC) coordinated or not to the accumulation of tokens in the Petri net simulation. Coordinated expression was considered when downregulated genes were targeted of upregulated miRNA and showed low accumulation of tokens after simulation. Since differential expression and token values were calculated considering the normalized readcount from the genes, the low token accumulation output was defined as less than the input token from the same gene and the interconnected gene. Coordinated expression was also evaluated as true in the inverse case, when upregulated genes were targeted of downregulated miRNA and resulted in high accumulation of output tokens. High token accumulation was defined as more than the input token from the same evaluated gene and its interconnected gene pair. Contradictory evaluation of gene pairs was defined when downregulated genes showed high accumulation of tokens and when upregulated genes showed low accumulation of tokens after simulation.

To interpret the miRNA knockout simulation, we considered the simulation of the miRNA knockout successful when the result output token from the target transcript was higher than the result output token in a simulation without miRNA knockout.

### 3.2 Visualization of the Petri nets

**Figure 4:**
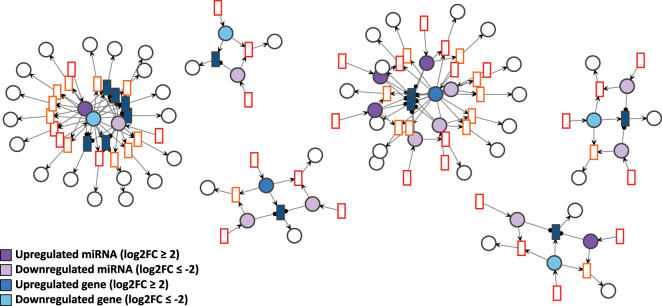
D4P Petri net visualized using the GEM layout. Genes and miRNA are the colored places, whilst transcript isoforms are the colored transitions. Labels and tokens are omitted for visibility.

**Figure 5:**
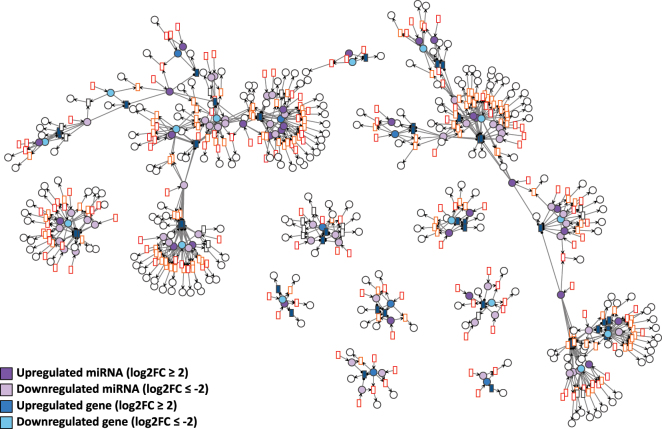
D9P Petri net visualized using the GEM layout. Genes and miRNA are the colored places, whilst transcript isoforms are the colored transitions. Labels and tokens are omitted for visibility.

**Figure 6:**
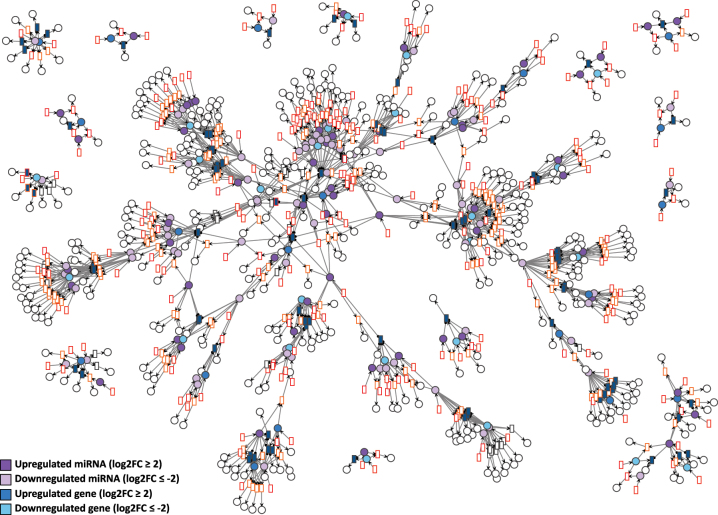
D15P Petri net visualized using the GEM layout. Genes and miRNA are the colored places, whilst transcript isoforms are the colored transitions. Labels and tokens are omitted for visibility.

Each GRN of the days D4P, D9P, and D15P was imported to VANESA as a biological network and further automatically transformed to a Petri net by the four defined transformation rules. All elements of the biological network (DNA, mRNA, and miRNA nodes and edges) were transformed successfully to Petri net elements (places, transitions, and arcs), while maintaining the proper interaction relationship between them, as shown in Figures 4–6.

To analyze the simulation of specific and relevant genes from the original biological networks, we focused on enhancing the visualization of key locations displaying the final expression simulation results.

### 3.3 Simulation of Petri nets and comparison with differential gene expression

To assess the significance of the modeling of the GRN, its transformation to a Petri net, and its simulation, we evaluated pairs of selected genes of interest, targeted by common miRNAs. Their relevance was addressed previously in the GRN of each day of cardiomyogenic differentiation D4P, D9P, and D15P [16]. The simulation results revealed that many gene pairs and common miRNAs exhibited token accumulation patterns consistent with the previously assessed differential gene expression analysis. In this case, simulations were categorized as coherent. However, some gene pairs accumulated more or fewer tokens than expected, indicating inconsistencies when compared to the differential expression analysis. These results were considered as incoherent.

#### 3.3.1 Coherent token accumulation with the differential expression analysis

The coherent token accumulation regarding the differential expression analysis was observed in all days of cardiomyogenic differentiation. It was seen not only with genes that shared miRNAs targets in common, but also in individual genes that did not interconnect in the networks.

**Figure 7:**
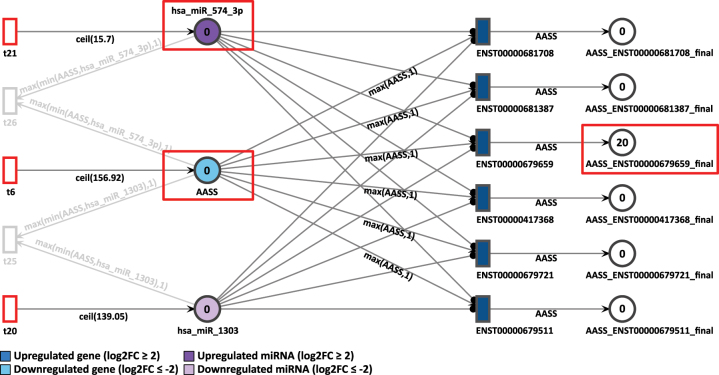
D4P Petri net, focus on the AASS gene after the simulation. Genes and miRNA are the colored places, whilst transcript isoforms are the colored transitions.

First, in D4P, the AASS gene was analyzed. It is also present in the D9P network. In the differential expression analysis, AASS is less expressed in D4P, presenting a log2FC of −2.99, as shown in Figure 7. All the transcripts from AASS gene, modeled as transitions, are targeted and inhibited by the miRNA hsa-miR-574-3p, which in turn is overexpressed with a log2FC of 1.67. Tokens in the place AASS and in the miRNA place are generated at a speed of 157 and 16 per time unit, respectively.

After 20 time units, all the AASS transcripts showed a result of zero generated tokens, except for the isoform ENST00000679659 with 20 tokens. The low accumulation of tokens in the simulation corroborates with the differential expression analysis in which AASS is less expressed, as it can be seen by the color code of light blue in the gene place.

**Figure 8:**
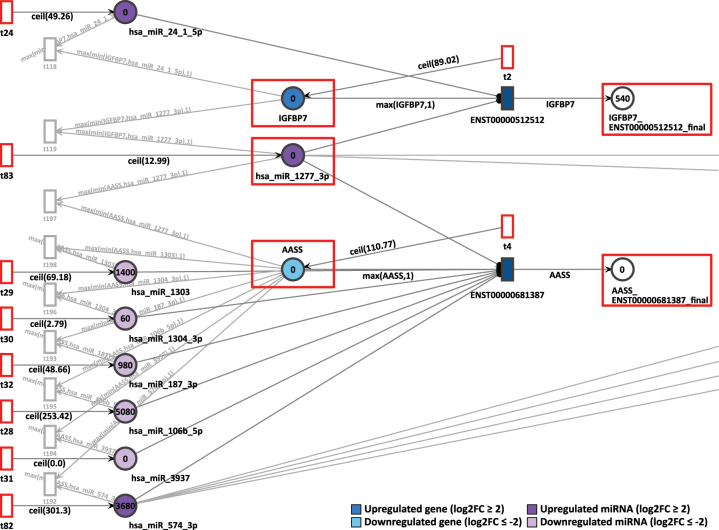
D9P Petri net, focus on the interconnected genes AASS and IGFBP7 through the common target miRNA hsa-miR-1277-3p. Genes and miRNA are the colored places, whilst transcript isoforms are the colored transitions.

Then, in the D9P network, the first connections between genes through common miRNA appears. The downregulated gene AASS was observed connected with IGFBP7, a gene most expressed in D9P with a log2FC of 3.09. Both are targeted by the miRNA hsa-miR-1277-3p, which is upregulated with an expression of log2FC equal to 2.12 (Figure 8). Tokens in the place AASS are generated with a speed of 111, in IGFBP7 with a speed of 90, and in the miRNA place hsa_miR_1277_3p with a speed of 13 per time unit.

In the final result of the simulation, it was possible to observe the overexpression of IGFBP7 through the accumulation of 540 tokens of its uniquely detected isoform ENST00000512512. The miRNA hsa-miR-574-3p, which inhibits AASS in the D4P Petri net (Figure 7), is also present with a constant token generation of 302. On day D9P of cardiomyogenic differentiation, it shows a slightly higher differential expression with a log2FC of 3.10. The accumulated token counts in both genes and isoforms align with the differential expression results. IGFBP7, which is more highly expressed, has more final tokens than AASS, which is less expressed in D9P.

**Figure 9:**
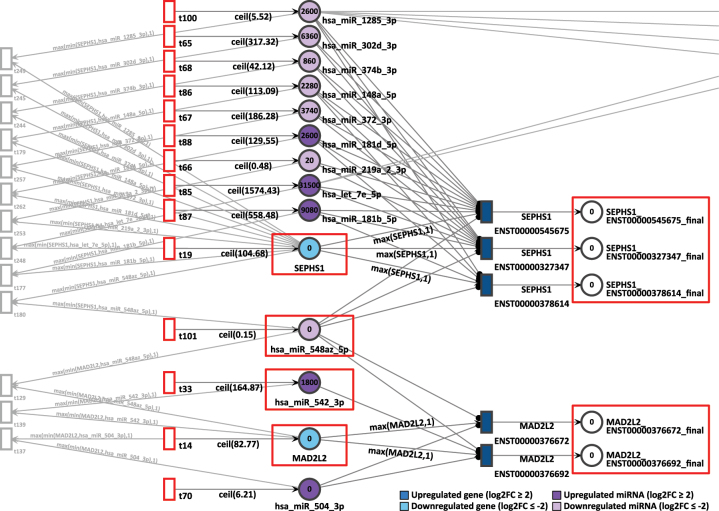
D9P Petri net, focus on the interconnected genes MAD2L2 and SEPHS1, interconnected through the common target miRNA hsa-miR-548az-5p. Genes and miRNA are the colored places, whilst transcript isoforms are the colored transitions.

The gene pair MAD2L2 and SEPHS1 showed coordinated token accumulation in both days, D9P and D15P. First, in D9P, both genes MAD2L2 and SEPHS1 are less expressed, with respective log2FCs of −2.20 and −3.00, whilst their token generation are 83 and 105 respectively, as shown in Figure 9. The miRNA they share, hsa-miR-548az-5p, has a constant generation of 1 token, with a low differential expression of log2FC equal to −4.59.

After 20 time units, none of the SEPHS1 transcripts produced any tokens, nor did any isoforms from MAD2L2. All isoforms from SEPHS1 and MAD2L2 are being targeted by highly expressed miRNA. The isoform ENST00000376692 is exclusively targeted by a highly expressed miRNA hsa-miR-542-3p, which receives constantly 165 tokens and has a log2FC of 1.47. Since both downregulated genes show zero accumulated tokens, their simulations were considered corroborating with differential expression analysis.

**Figure 10:**
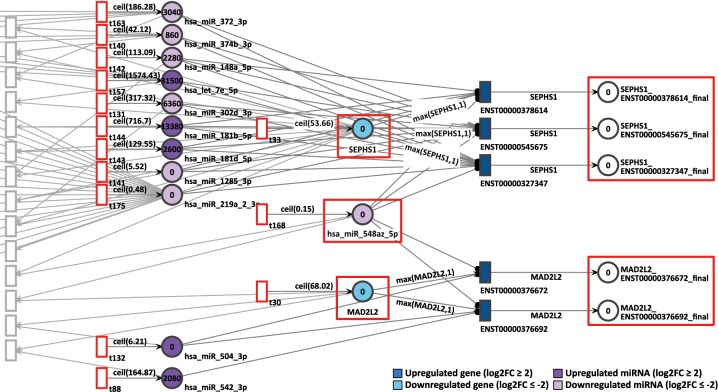
D15P Petri net, focus on the interconnected genes MAD2L2 and SEPHS1, interconnected through the common target miRNA hsa-miR-548az-5p. Genes and miRNA are the colored places, whilst transcript isoforms are the colored transitions.

The same gene pair is present in D15P, with the difference that MAD2L2 shows a lower generation of incoming tokens than in the previous network, with a constant generation of 69 tokens, as shown in Figure 10. The differential expression still remains low at D15P with log2FC of −2.43. The same pattern is found with the gene SEPHS1 as its constant token generation of 54 is almost half as many as before and also remains less expressed with log2FC of −3.95. The common miRNA hsa-miR-548az-5p that interconnects these genes presents the same values as in the previous network, with a constant token generation of 1 and log2FC of −4.59.

After 20 time units, none of the SEPHS1 transcripts produced tokens at the end, nor did the isoforms from MAD2L2. Repeating the pattern seen in D9P, here in D15P we also see both downregulated genes showing zero token accumulation. Therefore, the simulation results corroborate with the differential expression analysis.

#### 3.3.2 Incoherent token accumulation with the differential expression analysis

In the final days of cardiomyogenesis, some genes did not show an accumulation of tokens coordinated with the differential expression analysis. This means that either high token sum was seen in downregulated genes or low token sum in upregulated genes.

**Figure 11:**
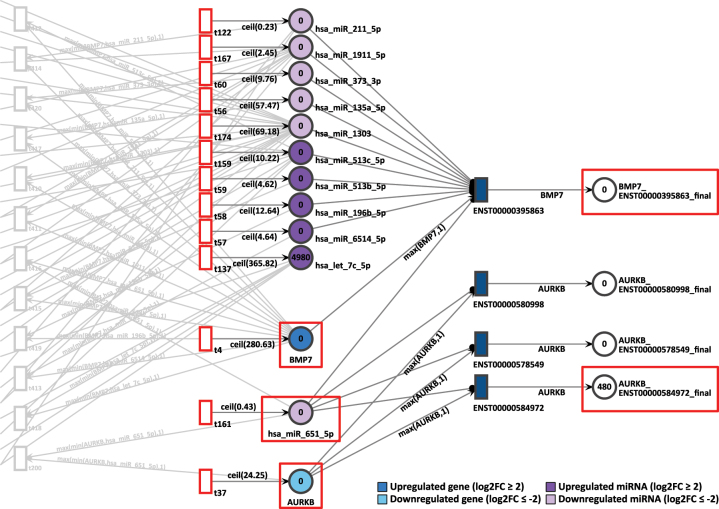
D15P Petri net, focus on the interconnected genes AURKB and BMP7, interconnected through the common target miRNA hsa-miR-651-5p. Genes and miRNA are the colored places, whilst transcript isoforms are the colored transitions.

In D15P we have observed the pair of genes BMP7 and AURKB. The gene BMP7 is overexpressed with a log2FC of 2.92 and a constant token generation of 281. On the other hand, the less expressed gene AURKB shows a log2FC of −2.20 and a constant token generation of 25, as seen in Figure 11. Both are targets of the common miRNA hsa-miR-651-5p, which in turn is strongly downregulated with a log2FC of −7.99 and a constant token generation of 1.

As a result, after 20 time units, only the isoform ENST00000584972 from AURKB generated 480 tokens in total, whereas the uniquely expressed isoform from BMP7 generated zero tokens. It would be expected that the most expressed gene BMP7 would show a significant amount of token accumulation. However, no tokens at all were accumulated after simulation by BMP7. In contrast, AURKB is less expressed than BMP7 in D15P and it presented more output tokens after simulation. Therefore, the accumulated token results do not corroborate with the differential expression results.

**Figure 12:**
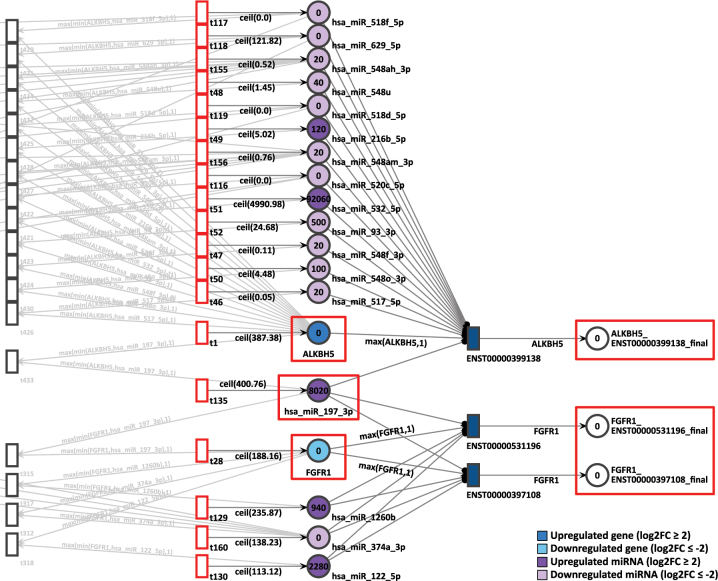
D15P Petri net, focus on the pair of genes ALKBH5 and FGFR1, interconnected through the common target miRNA hsa-miR-197-3p. Genes and miRNA are the colored places, whilst transcript isoforms are the colored transitions.

In the same D15P network, the pair of genes FGFR1 and ALKBH5 are sharing the miRNA hsa-miR-197-3p in common. While FGFR1 presents low expression with a log2FC of −2.06 and a constant generation of 189 tokens, ALKBH5 is overexpressed with log2FC of 2.03 and receives 388 tokens constantly (Figure 12). The hsa-miR-197-3p miRNA is also upregulated with a log2FC of 1.96 and a token generation of 401.

After 20 time units, neither isoform from FGFR1 generated any tokens. The zero token generation from downregulated gene FGFR1 corroborates with the differential expression reference. However, despite ALKBH5 being highly expressed in D15P, it also presented zero token accumulation in its uniquely expressed isoform. Therefore, for this pair of genes, the simulation results does not corroborate with the gene expression analysis.

#### 3.3.3 miRNA knockout simulation

**Figure 13:**
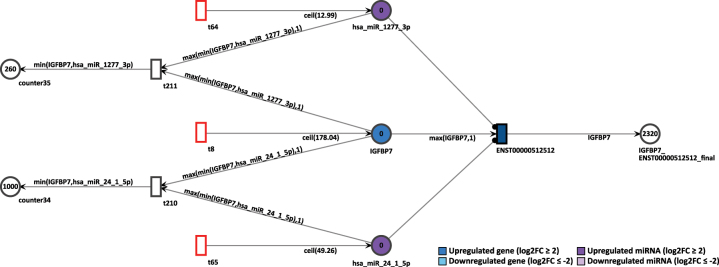
Simulation of the IGFBP7 gene in the D15P Petri net.

To simulate the knockout of miRNA, we observed the gene IGFBP7 in D15P. This gene was already present in the previous Petri net of D9P. First, we assessed the normal simulation of the gene, without removing the inhibition by the miRNAs. In D15P, IGFBP7 is upregulated with log2FC of 4.15, and a constant token generation of 179, as shown in Figure 13. Its uniquely expressed isoform ENST00000512512 is the target of two upregulated miRNAs, hsa-miR-1277-3p that shows log2FC of 2.12 and a constant token generation of 13, and hsa-miR-24-1-5p with log2FC of 3.86 and a constant token generation of 50.

**Figure 14:**
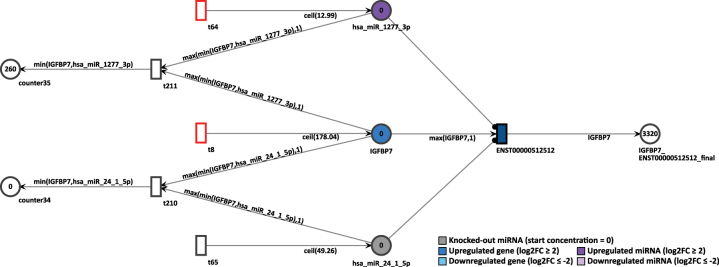
Simulation of the IGFBP7 gene after the miRNA hsa-miR-24-1-5p knockout in the D15P Petri net.

In normal settings, after 20 time units, the IGFBP7’s only transcript generated 2,320 tokens. Thus, the high token accumulation corroborates with the expression analysis, since IGFBP7 is upregulated. To simulate a miRNA knockout, the constant token generation of the miRNA hsa-miR-24-1-5p is reconfigured to zero by deactivating its feeding transition. After rerun of 20 step simulation, the accumulated output tokens of the transcript of IGFBP7 increased to 3,320 tokens, as shown in Figure 14. The increase of tokens from the targeted transcript is an expected outcome. Since miRNA regulation inhibits the target transcripts, if such miRNA is itself suppressed, then the targeted transcript can be expressed. Therefore, the knockout simulation corroborates with the inhibition of a miRNA.

## 4 Discussion

### 4.1 Modeling of GRNs as Petri nets

Complex biological systems can be investigated with more detail through the construction of networks in order to elucidate the interactions between the genes. Computational approaches to model said networks enhance their understanding by investigating a substantial amount of data to pinpoint the relevant gene regulations [19], 20]. Furthermore, an *in silico* oriented investigation through network modeling allows the simulation of gene expression manipulation prior to conducting *in vitro* experiments, which then can be focused on relevant predicted signaling [21], 22].

The software VANESA enables the intuitive modeling and visualization of biological networks, the modeling and simulation of Petri nets, and the rule-based transformation of biological networks to Petri nets.

Gene expression can be regulated by other means that were not accounted for during the construction of our GRNs, including lncRNAs and transcription factors (TFs). Through the presence of miRNA binding sites, lncRNAs can act as miRNA sponges, capturing the miRNAs and preventing interactions with their mRNA targets and thus hindering its inhibitory activity [63]. In this way, lncRNAs compete with other target transcripts, reducing the regulatory ability of miRNA on their original targets [63]. Therefore, lncRNAs could alter the simulation and construction of the present regulatory network. Previous work by our group has already shown that there is extensive lncRNA expression throughout the cardiomyogenesis differentiation of hESCs [14] and they can play an important role in the cardiomyocyte cell cycle [64].

Here, we have searched for miRNA regulations related to the APA modification, specifically on the 3′UTR of coding mRNAs, which is exclusive of mRNAs. As lncRNAs do not typically have a 3′UTR, they were initially excluded from the miRNA target identification. However, inclusion of known lncRNA target information, especially in the context of the heart development, could greatly improve our GRN models and corresponding Petri nets to consider this extra layer of regulation [64].

Other relevant molecules that were not investigated in our GRNs are TFs, which are regulatory elements that bind to specific DNA sequences, enabling their control of gene transcription rate [65]. TFs are known to be key players in gene expression and in processes such as stem cell development and differentiation [65]. Moreover, there are TFs that regulate more than one gene, which could be interesting to model in a network. In this manner, taking TFs into account in our GRNs could help to determine the activation strength of the genes present in the network. Consequently, the gene’s expression could be fine-tuned in the GRNs and generated Petri nets. These factors are also important for the regulation of cardiomyocytes proliferation [66] and stem cell pluripotency [67]. Therefore, their inclusion in this present network could greatly enhance the analysis. Other relevant post-transcriptional regulations, such as RNA half-life [68] and inhibition capacity of each miRNA molecule, were not modeled in our GRNs. However, we were able to illustrate the regulation of miRNAs on their targets in a more dynamic way than traditional GRNs.

### 4.2 Petri net simulations

The aim of a network simulation is to predict real data [21], 22], 25]. Therefore, it was expected that overexpressed genes would generate more result tokens than genes that were not highly expressed. Similar to a previous study which compared two factors between each other in order to validate the simulation [22], here, we have also selected relevant pairs of genes to evaluate the simulation result according to their differential expressions. We have selected the genes due to their miRNAs in common and to their relevance to cardiomyogenic differentiation.

Many genes that are relevant for cardiomyocyte differentiation showed coherent simulation results with expression analysis. In the first network, D4P, the downregulated gene AASS showed a low accumulation of tokens after simulation. Then, in the ninth day of differentiation, the gene IGFBP7 was observed interacting with AASS through a common miRNA, hsa-miR-1277-3p. In the final cardiomyocyte stage, IGFBP7 was not seen interacting with any other gene, apart from the miRNA. In both networks, D9P and D15P, the upregulation of IGFBP7 could be validated by the higher accumulation of tokens in its unique transcript. In fact, IGFBP7’s only expressed transcript generated more than 2,000 tokens in D15P. These network results corroborate previous work that confirmed the expression of IGFBP7 *in vitro* in cardiomyocytes [69]. It is interesting how the same set of genes are present in each stage of cardiomyogenesis, despite showing different interactions.

In another example, the downregulated gene SEPHS1, targeted by 9 miRNAs, generated zero tokens after the simulation, not only in the D9P network, but also in D15P. In both networks, it was interacting with MAD2L2 through the common miRNA hsa-miR-548az-5p. MAD2L2 also showed zero token accumulation after simulation. The pattern of token accumulation of both genes was compatible with their low expression in the last two stages of cardiomyogenesis. This observation also aligns with previous work where the deficiency of SEPHS1 is associated with cardiac development [70]. Furthermore, SEPHS1 was targeted by two interesting miRNAs: first, the highly expressed hsa-let-7e-5p, a miRNA from the let-7 family, which is essential for cardiomyocyte maturation [71], 72] and second, the hsa-mir-302d-3p, a miRNA which is known to participate in the proliferation of stem cells that derive into cardiomyocytes [73].

However, there were simulation results which did not match what was found in the differential expression. In D15P, the downregulated gene AURKB generated more output tokens than the upregulated gene BMP7. BMP7 is known to be important for cardiomyogenic development [74] and together with FGFR1 it assists in differentiation to cardiomyocytes [75]. Meanwhile, AURKB is expected to be less expressed in differentiated cardiomyocytes, since its expression is associated with their proliferation [76].

Also in the D15P network, the gene FGFR1 showed inconsistent simulation results while interacting with ALKBH5 through a common miRNA. Even though FGFR1 was less expressed and showed zero token accumulation, its interacting gene ALKBH5 showed contradictory results. Despite being upregulated in D15P, the only transcript from ALKBH5 accumulated zero output tokens. The relationship of FGFR1 and ALKBH5 through the sharing of the miRNA hsa-miR-197-3p has been observed previously, as seen in the experimentally validated database StarBase [77]. Additionally, it is known that suppression of FGFR1 is important for cardiomyogenic differentiation [75]. The gene ALKBH5 plays an important role in epigenetic modification by encoding for a demethylase that modifies N(6)-methyladenosine (m6A) on RNA [78]. It is an essential process even in cardiomyogenesis, as its overexpression induces cardiac regeneration and cardiomyocyte proliferation [79]. Since we have investigated gene expressions, chromatin modifications were not taken into account. Thus, in the context of cardiomyogenesis, this gene pair is a good candidate for refinement of the Petri net models to achieve congruent simulation results.

In this manner, rather than expecting all the simulation results to be in agreement across the entire network, the interpretation of simulation results should pay attention to the groups of genes that are relevant to each other [22], 25]. Other work has also interpreted the Petri net sublevels or clusters, evaluating groups of genes that would integrate a specific pathway [37]. Furthermore, it is also important to note that supplementary experiments are needed to confirm the simulation results of specific pairs of genes [22]. Many works have focused on only a selected regulatory pathway that comprises a limited number of genes and/or cytokines [41], 44]. In contrast, we have generated three Petri nets with 64, 329, and 543 places each. The largest network, D15P, consists of 543 places, 543 transitions, and 1,471 edges. A small margin of incongruence of the simulation results is expected due to the size of the models, as fewer nodes have the advantage of being more accurate [20]. In other words, the more complex the gene regulatory network, the more difficult it is to build an accurate model [20]. In addition, the concentrations of the genes and miRNAs were obtained from bulk RNA-sequencing, which evaluates the behavior of a population of cells. Variations and fluctuations are inherent to biological diversity, which can also hinder the accuracy of the model [20].

The incongruence of the models with *in vitro* experiments can also indicate missing information in the known biological network. This is a hypothesis that should be investigated further, especially in the case of ALKBH5 and FGFR1 due to their notorious role in cardiomyogenesis [75], 79]. Interesting to notice is that ALKBH5 is an upregulated gene in the GRN of D15, even though it is targeted by 13 miRNAs, two of them highly expressed. There could be a missing key player that suppresses inhibition of these miRNAs. The work of Bonzanni and collaborators showed that the Petri net modeling of the haematopoietic differentiation was indicating a missing repression of a gene, which was not accounted for in experimental evidence [43]. Thus, due to some contradictory results obtained by the simulations, it could indicate that regulatory elements might also be missing in our model. Other processes, such as molecular degradation, might also play a role, especially in the context of RNA half-life [68]. This case has been considered in a Petri net model of stem cell self-renewal network [44] and could be investigated further.

Finally, we have also performed a miRNA knockout simulation that targets the gene IGFBP7 and its uniquely expressed isoform ENST00000512512. Since the miRNA inhibits the expression of their target gene, it is predicted that its absence will lead to a higher expression of its target. As expected, the absence of inhibition of the hsa-miR-24-1-5p miRNA resulted in a greater number of tokens generated by the IGFBP7 transcript. As already mentioned, the expression of IGFBP7 in cardiomyocytes was previously confirmed *in vitro* [69]. The miRNA knockout is an *in silico* approach that could contribute to the investigation of these key players in the context of cardiomyogenesis. Moreover, we have chosen to simulate a knockout situation, but other works have successfully used anti-miRNA approaches to suppress the miRNA expression in the Petri net model [45]. It is also possible to increase the concentration of any node in the biological network, modeling the overexpression of a gene, transcript, or miRNA. A higher start concentration of either IGFBP7 or target miRNAs could show different simulation outcomes. This illustrates the possibilities of gene expression manipulation in our cardiomyogenic differentiation Petri nets.

### 4.3 Application of the model

The systems biology approach of transforming a biological network into a computational model for simulation and analysis can have different aims, from understanding the processes to predict behaviors of the elements due to modifications [20]. In the medical field, this can be translated into a variety of objectives, recently thoroughly reviewed [80]: first, models can elucidate dysfunctional processes in disease and cancer [20], 25], 37], second, disease models can reveal the effect of drugs and treatments [46], and third, models can be used to predict apoptosis [41] or mortality [81]. However, before investigating disrupted gene regulation in the context of disease, it is essential to understand how the steady-state is supposed to be. Thus, models of well-studied and established signaling pathways need to be thoroughly constructed and validated [36], 40] before they can be disrupted. In regenerative medicine, stem cells stand out in the field by their intrinsic capacity of self-renewal and differentiation [6]. Thus, they could be used to recolonize, regenerate, and/or repopulate damaged tissues [3]. The differentiation process of stem cells has been modeled as Petri nets before, particularly in angiogenesis and hematopoiesis [43], 44], 82]. To our knowledge, however, Petri net modeling in the context of cardiomyogenesis has not been done, yet.

Prior, we have shown that not only genes, but also miRNAs and their specific targets over the alternative polyadenilated isoforms played an important role in the differentiation process of stem cells into cardiomyocytes [16]. Following these findings, we have transformed the GRNs to Petri nets, allowing further applications:

First, it enables investigating the functional orchestration of transcripts that successfully differentiate stem cells into cardiomyocytes from a holistic point of view. New interactions between genes through miRNAs can be observed. Possible role of known genes [82] or missing regulation elements can be hypothesized, due to unexpected simulation results [43].

Second, it facilitates manipulation of gene expression, in the case of knockout or overexpression. This proves a valuable tool for those interested in cardiomyogenesis and/or the miRNA regulation over alternative isoforms. Instead of immediately studying the cardiomyocyte differentiation process *in vitro*, one can use the modeled GRNs and generated Petri nets to optimize concentrations, knockout miRNAs or even to overexpress genes. Not only prediction of *in vitro* results is expected, but also various benefits for the research, such as reduction of animal usage, reagents, financial costs, and time [20], 40]. Experimental design can be planned ahead using the computational models before *in vitro* validation.

Finally, consequences of the mentioned investigations can contribute to the field of regenerative medicine. A better understanding and control over how stem cells differentiate to cardiomyocytes is a step forward towards the application of these cells in the context of cardiovascular diseases [4].

### 4.4 Improvement of the model

Due to some inconsistent simulation results, as previously stated, we believe that our model can still be improved in a number of ways. Quantification at the transcript level by long-read RNA sequencing [83] would enable more precision in the expressed isoform identification and consequently improve the simulation results. Normalization of the read counts for gene length in addition to library size could reduce quantification noise [84]. The evaluation of simulation results could be validated with significance analysis of concentration changes [45], 46] or considering constant transition and place invariants [37]. The inclusion of new categories of nodes, such as TFs and lncRNAs, to the models could help to fine-tune the representation of transcript expression and miRNA inhibition. VANESA already provides modeling of lncRNAs and TFs in biological networks, which allows the implementation of these elements to improve the network. The miRNA-sponge activity of lncRNAs and regulatory effect of TFs could be represented in the set of transformation rules in VANESA. Thus, it is possible to enhance the presented initial GRNs resulting in more complex and sophisticated models.

Given the simplicity of the network compared to the complex cellular environment, which includes several other factors influencing post-transcriptional regulation, it would be interesting to enhance the Petri net models by such factors. One potential approach is to incorporate probabilistic behavior by using stochastic transitions, a methodology that has been successfully implemented in a biological Petri net in the past [22]. In detail, a probability distribution is assigned to each stochastic transition to determine its delay. Thus, each transition has to wait a random amount of time until it can fire again, instead of a constant amount of time. Assigning random delays to the transitions involved in the inhibition of miRNAs could help to refine their processes.

Further, a GRN could represent continuous behavior, such as transcription rates given as functions, once sufficient data about the processes is available. Such a GRN could then be transformed to a hybrid Petri net reflecting not only discrete but also continuous behavior. VANESA supports transformation to, modeling of, and simulation of hybrid Petri nets with stochastic transitions given its implemented xHPN formalism.

## 5 Conclusions

The use of Petri nets proved to be a good strategy for modeling and simulating the GRNs previously constructed through a holistic point of view. Interactions between genes and miRNAs identified in *in silico* experiments could be observed in the networks and were usually in agreement with the simulation results. Here, we have modeled post-transcriptional GRNs with a specific miRNA regulation on APA isoforms, transformed them automatically into Petri nets, and simulated a miRNA knockout, providing a dynamic point of view of the process of cardiomyogenic differentiation of hESCs.

Naturally, there is still potential for improvements. The comparison between the model’s prediction with the experimental data can indicate whether the model is adequate or needs to be revised if incoherent results are found [23]. The models representing post-transcriptional regulation during cardiomyogenic differentiation align with the initial differential expression analysis and the existing literature. However, as demonstrated here, the effectiveness depends on the specific genes being evaluated. The simulation results were best interpreted regarding relevant pairs of genes and shared miRNAs whose interaction have already been observed experimentally. This *in silico* analysis allowed us to understand biological phenomena faster than linear and experimental *in vitro* methods, since they require a greater amount of time and resources to analyze a large number of gene interactions compared to computational models [20].

The modeling of the post-transcriptional GRNs of differentiating cardiomyocytes helps to elucidate the dynamic interplay between the transcripts during cardiomyogenesis, considering not only their concomitant interactions but also the simulation of their expressions. Prospects for these models are varied, such as the inclusion of additional elements, e.g., lncRNAs, TFs, transcript-level quantification, and *in vitro* validation of the simulated miRNA knockout.

## 6 Supplementary material

All of the necessary data to reproduce the figures of this article are publicly available in the following resource: https://doi.org/10.5281/zenodo.15190369. The biological networks and the Petri nets of all the days of cardiomyogenesis are available, as well as the set of rules needed to transform the GRN to Petri nets, all the simulation results and the adapted miRNA knockout network. Additional information on how to install VANESA, visualize the GRN, transform the Petri nets and upload the simulation results is provided.

## Supplementary Material

This article contains supplementary material (https://doi.org/10.5281/zenodo.15190369).
